# The efficacy of rituximab in pediatric patients with steroid-dependent or frequent relapsing nephrotic syndrome due to MCD or FSGS

**DOI:** 10.3389/fmed.2026.1763615

**Published:** 2026-04-23

**Authors:** Yue Xi, Ying Liang, Zhi Chen, Lei Lei, Lan Mi, Xiaoyu Tian, Liuyu Sun, Qiang Sun, Nan Zhou

**Affiliations:** Department of Nephrology, Beijing Children’s Hospital, Capital Medical University, National Center for Children’s Health, Beijing, China

**Keywords:** children, frequent relapsing nephrotic syndrome, minimal change disease, primary focal segmental glomerulosclerosis, rituximab, steroid-dependent nephrotic syndrome

## Abstract

**Background:**

Rituximab has been shown efficacy in reducing relapses among patients with steroid-dependent or frequently relapsing nephrotic syndrome (SDNS/FRNS), primarily due to minimal change disease (MCD) or focal segmental glomerulosclerosis (FSGS). However, whether its efficacy differs between these two histological subtypes remains unclear.

**Methods:**

A retrospective cohort study included 42 pediatric SDNS/FRNS patients with biopsy-proven MCD or FSGS. Clinical outcomes, including remission rates and relapse frequency were assessed.

**Results:**

The median age at diagnosis of the 42 patients was 5.1 years (IQR: 2.9–7.4), and 28 (67%) patients were male during the 6-year follow-up. Each patient received a median of 4 (IQR: 3–5) infusions. All patients achieved clinical remission, with 90.5% attaining complete remission. Relapse times decreased to 35.7% within 1 year after the first rituximab treatment, from a median of 2 relapses to 0. No significant difference in relapse rates was observed between patients receiving 3–4 doses versus 1–2 doses during the initial course. Histologically, 36 patients (85.7%) had MCD and 6 (14.3%) had FSGS. In the FSGS group, the 96-month relapse-free survival was higher compared to MCD controls (2/6 [33.3%] vs. 15/36 [41.7%]; *p* < 0.01; HR, 0.13; 95% CI, 0.05–0.35). Rituximab was well tolerated.

**Conclusion:**

Rituximab is effective for SDNS/FRNS children, whatever MCD or FSGS. These findings support repeated rituximab use in SDNS/FRNS. However, given the small sample size of the FSGS subgroup, these findings should be interpreted with caution and require validation in larger, multicenter studies.

## 1 Introduction

Primary nephrotic syndrome (PNS) is a prevalent glomerular condition among children, with occurrence rates ranging from 2 to 16.9 per 10 million across various races and areas (1). Among its diverse pathological subtypes, minimal change disease (MCD) and focal segmental glomerulosclerosis (FSGS) pose significant challenges, often resulting in steroid-dependent (SD) or frequently relapsing (FR) nephrotic syndrome (SDNS/FRNS) (2).

Corticosteroid treatment is fundamental for the first occurrence (2). Nevertheless, following corticosteroid treatment, only 30% of patients with steroid-sensitive nephrotic syndrome (SSNS) remain relapse-free for a year, and nearly half progressing to SDNS/FRNS (3–5). The guidelines from Kidney Disease: Improving Global Outcomes (KDIGO) recommend considering the use of steroid-sparing agents like calcineurin inhibitors (CNI), cyclophosphamide, or mycophenolate mofetil (MMF) in such cases (2). Nevertheless, these immunosuppressants have several adverse effects, including increased infection risk, diabetes, and renal toxicity, necessitating the exploration of novel therapeutic approaches.

Rituximab, a monoclonal antibody targeting CD20, has proven effective in decreasing relapses in patients with SDNS/FRNS (2, 3). In multiple randomized controlled trials, rituximab was employed to either stop or decrease the use of corticosteroids and steroid-sparing agents (SSAs) in children with SDNS (6–8). However, there is still a lack of studies on the specific dosing regimens, the efficacy of rituximab, and whether there are differences among different pathological patterns.

In this retrospective study, we aimed to evaluate the efficacy of rituximab in children with MCD or primary FSGS who presented with SDNS or FRNS. In addition, we intended to compare the treatment responses to rituximab between patients with MCD and primary FSGS.

## 2 Methods

### 2.1 Patients

A total of 42 patients with biopsy-proven MCD or primary FSGS and SDNS/ FRNS received rituximab treatment at Beijing Children’s Hospital from January to December 2024. Their clinical data were examined retrospectively at the time of kidney biopsy and during each follow-up appointment. The inclusion criteria: (1) diagnosed as MCD or primary FSGS by kidney biopsy; (2) defined as SDNS/FRNS during follow-up; (3) followed for at least 12 months after the first course of rituximab; and (4) At the initial administration of rituximab, all patients were steroid sensitive. The exclusion criteria: (1) patients with active infections: including active tuberculosis, hepatitis B virus (HBV) infection et al.; (2) patients with immunodeficiency disorders; and (3) patients with malignancies, including solid organ tumors or hematologic malignancies.

### 2.2 Rituximab regimens

Rituximab was administered intravenously at a dose of 375 mg/m^2^ per infusion (maximum 500 mg). The first course consisted of one to four infusions, initiated after the patient had achieved remission. The number of rituximab infusions in the initial course was determined by the attending physician according to the patient’s clinical condition, treatment response, and B-cell depletion status. If two infusions were separated by more than 4 weeks, they were considered to belong to different courses. The cumulative dose referred to the entire amount of rituximab administered throughout all treatment sessions. The duration of concomitant immunosuppression was tailored to each individual and defined as use of one or more agents, such as corticosteroid, MMF, and/or CNI. In patients who maintained stable remission, tapering of MMF and/or CNI was generally initiated approximately 6 months after rituximab administration. To prevent infusion reactions, all patients received a single standard dose of dexamethasone 30 min prior to rituximab infusion, along with intramuscular promethazine.

During follow-up, the need for additional rituximab infusions was determined based on the recovery of circulating B cells. Retreatment was administered at the same dose (375 mg/m^2^, max 500 mg) to maintain B-cell depletion, defined as a circulating CD19^+^ B-cell count below 5 cells/μL. However, if relapse occurred or B-cell reconstitution was detected (CD19^+^ B cells >5 cells/μl), additional rituximab infusions were administered to restore B-cell depletion and maintain remission for 1–2 years. The schedule for administering rituximab was modified based on the specific characteristics of each patient. All patients received trimethoprim-sulfamethoxazole for pneumocystis jirovecii pneumonia (PCP) prophylaxis at the time of the first rituximab infusion. Since rituximab was first administered, glucocorticoids were either gradually reduced or entirely discontinued. Each patient participated in follow-up sessions scheduled every 1 to 3 months. At the start and during each visit, laboratory tests were conducted, which included 24-h urine protein, serum albumin, creatinine, eGFR, and the quantity of circulating B cells. Rituximab-related adverse events were assessed both during drug infusion and throughout the entire follow-up period.

### 2.3 Treatment responses and kidney outcomes

According to the 2025 KDIGO guidelines, the treatment effect was categorized as complete remission (CR), partial remission (PR), or no remission (9). Relapse was defined as a uPCR ≥2 mg/mg (≥200 mg/mmol) or ≥3 + protein on a urine dipstick for three consecutive days. SD was defined as two consecutive relapses during steroid therapy or within 2 weeks of ceasing therapy. FR was defined as experiencing two or more relapses within 6 months following the initial response, or having three or more relapses over any 12-month period. Rituximab was administered as a second- or third-line therapy in patients with SDNS/FRNS. Neutropenia and agranulocytosis were defined as absolute neutrophil count <1.5 × 10^9^ and 0.5 × 10^9^ per liter, respectively. Hypogammaglobulinemia was defined as serum IgG levels below the age-adjusted lower limit of the normal range. Hypogammaglobulinemia was graded according to serum IgG levels as mild (4–6 g/L), moderate (2–4 g/L), and severe (<2 g/L). Intravenous immunoglobulin replacement therapy was administered when IgG levels decreased to <4 g/L or when clinically indicated.

To assess kidney outcomes, kidney dysfunction was defined as a decline of eGFR >50% from baseline. eGFR was calculated using the modified Schwartz formula (10).

### 2.4 Statistical analysis

Statistical analyses were performed using SPSS version 25.0 (SPSS Inc., Chicago, IL, United States). Normality of continuous variables was assessed with the Shapiro–Wilk test. Normally distributed data were presented as mean ± standard deviation and compared using the independent samples *t*-test, non-normally distributed data were presented as median (interquartile range) and compared using the Mann–Whitney U test. Categorical variables were expressed as frequencies and percentages and compared using the chi-square test or Fisher’s exact test, as appropriate. The relapse rate was defined as the proportion of patients experiencing at least one relapse during the specified follow-up period, and the Wilcoxon signed-rank test was used to compare median annual relapse frequency before and after rituximab treatment. Kaplan–Meier survival analysis was used to estimate 12-month and 96-month relapse-free survival rates, with differences between histological subgroups (MCD vs. FSGS) assessed by the log-rank test and hazard ratios (HR) derived from Cox proportional hazards models. Relapse rates at 6 months and 1 year were compared between patients receiving 3–4 versus 1–2 doses during the first course using the chi-square test. All *p*-values are two-tailed, and *p* < 0.05 was considered statistically significant.

## 3 Results

### 3.1 Patient baseline information

As shown in Table 1, 42 patients with SDNS/FRNS were included and received rituximab treatment. Among these, 36 (85.7%) patients had MCD and 6 (14.3%) patients were diagnosed with primary FSGS. The cohort consisted of 28 (66.7%) males and 14 (33.3%) females, with a median age of 5.1 (IQR, 2.9–7.4) years. The time from the first rituximab therapy to the last follow-up was 22.5 (13, 28.3) months, and the overall follow-up time was 72 (44.5, 96.8) months.

**Table 1:** Baseline clinical parameters of children receiving rituximab therapy, who presented with SD/FR nephrotic syndrome due to MCD or FSGS.

Clinical information	*N* = 42
Age (years)	5.1 (2.9, 7.4)
Gender (male), *n* (%)	28 (66.7)
Body mass index, kg/m^2^	21.03 ± 5.75
Kidney pathology	
FSGS, *n* (%)	6 (14.3%)
MCD, *n* (%)	36 (85.7%)
Proteinuria (mg/d)	79 (43.75, 316.9)
Serum albumin (g/L)	34.51 ± 8.27
Serum total cholesterol (mmol/L)	6.11 ± 2.48
Serum creatinine (μmol/L)	36.54 ± 7.49
eGFR (ml/min/1.73m^2^)	135.4 ± 21.87
B-lymphocyte count (cells/μL)	668 (481, 1,298)
Disease course before rituximab therapy (months)	46 (21.75, 74.25)
Relapse times within 6 months before rituximab therapy	1.5 (1, 2)
Relapse times within 1 year before rituximab therapy	2 (2, 3)
Patients with more than two relapses, *n* (%)	42 (100%)
Concomitant steroid/immunosuppressive therapy at the first course of rituximab therapy, *n* (%)	
Glucocorticoids (MCD/FSGS)	40 (95.2%) (34/6)
Cyclosporine (MCD/FSGS)	4 (9.5%) (4/0)
Tacrolimus (MCD/FSGS)	12 (28.6%) (10/2)
Mycophenolate mofetil (MCD/FSGS)	13 (31%) (12/1)

Continuous variates in non-normal distribution are presented as median (interquartile range); continuous and normally distributed variables are presented as mean ± SD; categorical variables were presented as *n* (%); MCD, minimal change disease; FSGS, focal segmental glomerulosclerosis.

Before starting rituximab treatment, all patients had received glucocorticoids in combination with a range of immunosuppressants, such as cyclosporine, tacrolimus, MMF, and cyclophosphamide. The median duration of previous treatment was 46 (21.75, 74.25) months, with patients experiencing a median of 2 (2, 3) relapses within 1 year before rituximab therapy. At baseline, 20 (47.6%) patients presented with SD nephrotic syndrome, and 22 (52.4%) patients had FR nephrotic syndrome. Among the entire cohort, 4 patients received rituximab within the first year after diagnosis, with two classified as FRNS and two as SDNS. At the time of the first infusion, the median eGFR was 135.4 ± 21.87 mL/min/1.73m^2^.

### 3.2 Overall rituximab treatment efficacy and B-cell response

**Figure 1:**
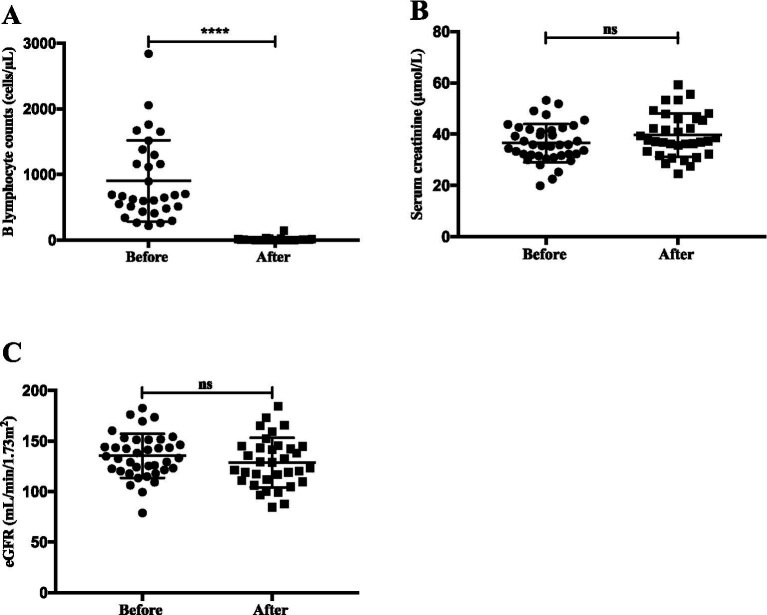
Comparisons in children with SD/FR nephrotic syndrome due to MCD or FSGS, before and after rituximab treatment: **(A)** B lymphocyte count; **(B)** serum creatinine; **(C)** eGFR.

All 42 patients received rituximab infusions, with a median of 4 (3, 5) infusions per patient, at a frequency of 2.5 (1.6, 3) infusions per year (Table 2). Rituximab treatment led to a significantly reduction in B lymphocyte counts compared to baseline [3 (0, 6.5) vs. 668 (481, 1,298) cells/μl, *p* < 0.0001] (Figure 1). At the initial administration of rituximab, all patients were steroid sensitive. Following rituximab therapy, all patients achieved clinical remission. No patient progressed to steroid-resistant nephrotic syndrome during follow-up. Serum creatinine (*p* = 0.10) and eGFR (*p* = 0.22) remained stable throughout treatment (Figure 1).

**Table 2:** Rituximab treatment response and follow-up data of children with SD/FR nephrotic syndrome due to MCD or FSGS.

Parameters	*N* = 42
Follow-up time from rituximab (months)	22.5 (13, 28.3)
Overall follow-up time (months)	72 (44.5, 96.8)
Rituximab dose each time (375 mg/m^2^, max 500 mg)	42 (100%)
Rituximab infusion frequency (times/year)	2.5 (1.6, 3)
Rituximab infusion times at the first course (times)	2 (1, 2.25)
Total times of Rituximab infusion during all the courses	4 (3, 5)
Treatment responses, *n* (%)	
Complete remission (MCD/FSGS)	27 (64.3%) (22/5)
Relapse (MCD/FSGS)	15 (35.7%) (14/1)

Continuous variates in non-normal distribution are presented as median (interquartile range); categorical variables were presented as *n* (%); Follow-up time from rituximab: The time from the first rituximab therapy to the last follow-up; MCD, minimal change disease; FSGS, focal segmental glomerulosclerosis.

**Figure 2:**
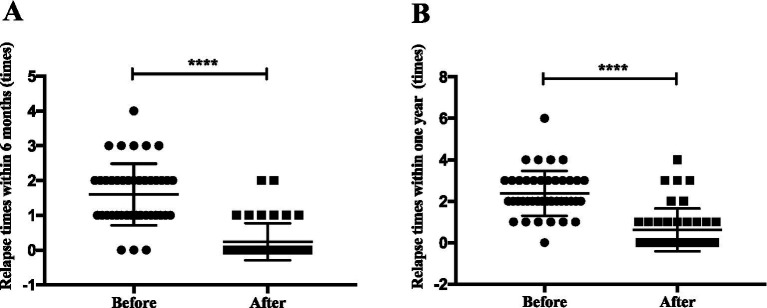
Relapse times comparisons in children with SD/FR nephrotic syndrome due to MCD or FSGS, before and after rituximab treatment. **(A)** 6 months before and after rituximab treatment; **(B)** 1 year before and after rituximab treatment.

**Figure 3:**
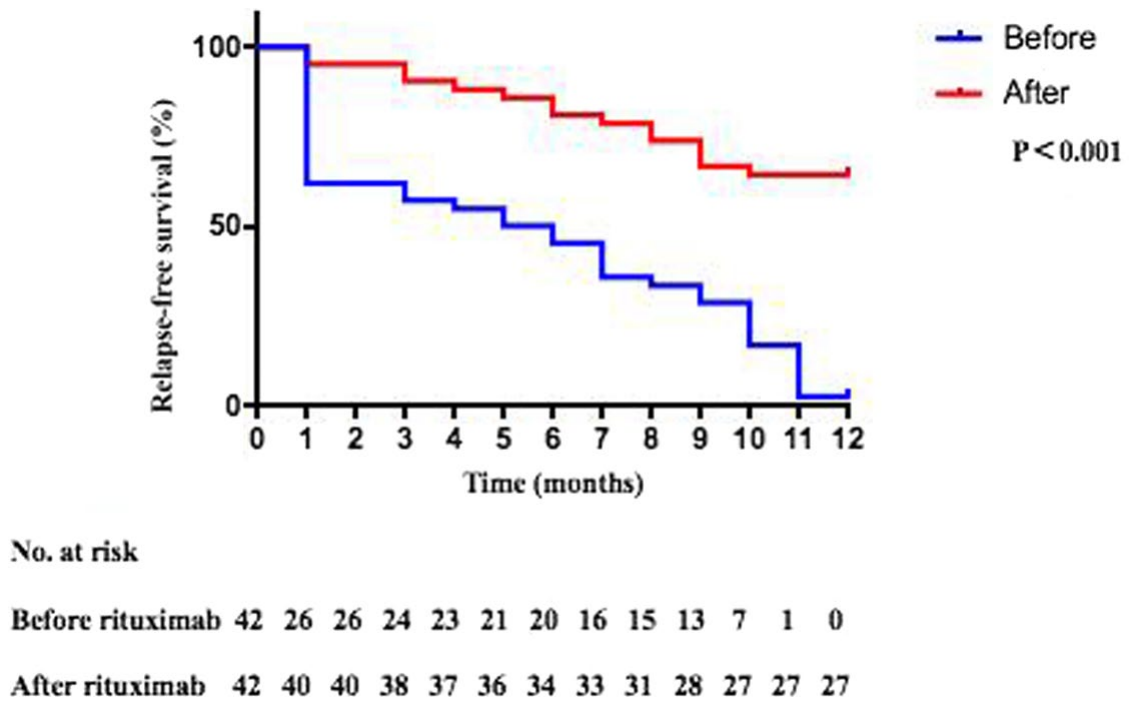
Comparisons of 12-month relapse-free survival rates in children with SD/FR nephrotic syndrome due to MCD or FSGS, before and after rituximab treatment.

Within 6 months after rituximab treatment, the relapse rate among SDNS/ FRNS patients decreased to 19% (8 of 42 patients). Within 1 year after rituximab treatment, the relapse rate among SDNS/ FRNS patients decreased to 35.7% (15 of 42 patients). The median frequency of relapse reduced from 2 (2, 3) relapse per year to 0 (0, 1) (*p* < 0.0001) (Figure 2). The 12-month relapse-free survival rate was significantly higher in patients after rituximab therapy compared with those before rituximab therapy (Figure 3).

### 3.3 Impact of initial rituximab dosing on relapse

The number of patients experiencing recurrence decreased progressively with increasing rituximab infusions during the first course (Table 3). There were 10 patients received 3–4 doses of rituximab at the first course. The median dosing interval was 8 days (IQR: 4–9.25). There was no significant difference in the relapse times at 6 months and 1 year between patients who received 4 doses of rituximab and those who received 1–3 doses in the first course (Supplementary Figure S1). Similarly, there was no significant difference in the relapse times between patients who received 3–4 doses of rituximab and those who received 1–2 doses in the first course (Supplementary Figure S1).

**Table 3:** Recurrence after the first course of rituximab treatment with different infusion times in pediatric patients with SD/FR nephrotic syndrome due to MCD or FSGS.

Rituximab infusion times at the first course (times)	Number of patients with recurrence, *n* (%) (MCD/FSGS)	First recurrence interval after Rituximab therapy (months)
1	9 (21.4%) (7/2)	7 (3, 16.6)
2	7 (16.7%) (7/0)	6 (4, 9)
3	1 (2.4%) (1/0)	8
4	0	–

Continuous variates in non-normal distribution are presented as median (interquartile range); First recurrence interval after rituximab therapy: the interval between the first recurrence time after rituximab therapy and the time of the first dose of rituximab.

### 3.4 Histology-specific responses

**Figure 4:**
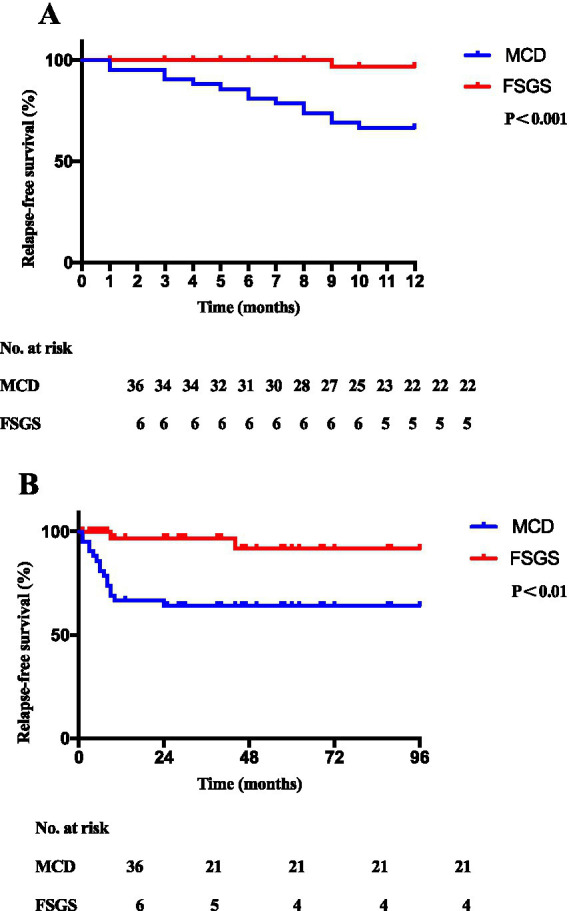
Probability of the 12-month **(A)** and 96-month **(B)** relapse-free survival in the FSGS group compared to MCD controls.

Despite rituximab treatment, 15 patients (14 with MCD and 1 with FSGS) experienced relapse within 1 year after rituximab treatment. The annual relapse rate was 38.9% (14 of 36 patients) in patients with MCD, and 16.7% (1 of 6 patients) in FSGS patients treated with rituximab. There was no difference in cumulative dose and course of rituximab between MCD and FSGS patients (Supplementary Figure S2). Cox regression model, the 12-month relapse-free survival was significantly higher in the FSGS group compared to MCD controls (1/6 [16.7%] vs. 14/36 [38.9%]; *p* < 0.001; HR, 0.07; 95% CI, 0.03–0.20) (Figure 4). The 96-month relapse-free survival in the FSGS group was also higher than the MCD control (2 of 6 [33.3%] vs. 15 of 36 [41.7%]; *p* < 0.01; HR, 0.13; 95% CI, 0.05–0.35) (Figure 4). Compared to MCD patients, the relapse times of FSGS patients was no difference within 6 months (*p* = 0.34) and within 1 year (*p* = 0.33) after the first course of rituximab treatment (Supplementary Figure S3).

All relapsed patients had B cells counts exceeding 5 cells/μl, with a median count of 556 (196, 624) cells/μl. All of these patients subsequently received additional rituximab treatment, and achieved complete remission once again.

### 3.5 Reduction of concomitant immunosuppressive therapy

Following initiation of rituximab, concomitant immunosuppressive medications were gradually reduced and tapered over time. The mean tapering time was 7.6 months for tacrolimus, 9.2 months for cyclosporine, and 8.7 months for mycophenolate mofetil. During follow-up, 21 patients (72.4%) were able to completely discontinue these agents, including 8 cases of tacrolimus, 3 cases of cyclosporine, and 10 cases of mycophenolate mofetil. Before rituximab treatment, patients required a median prednisone dose of 0.59 (IQR 0.27–0.98) mg/kg on alternate days to maintain remission. Following rituximab therapy, the steroid dose was significantly reduced to 0 (IQR 0–0) mg/kg during follow-up. In addition, 38 patients (90.48%) were able to completely discontinue prednisone after rituximab treatment.

### 3.6 Safety and adverse events

The therapy with rituximab was well-tolerated, with no severe or life-threatening side effects reported (Table 4). A total of 7 adverse events were observed in 5 (11.9%) patients. Among all treatment episodes, hypogammaglobulinemia (71.4%, 30/42) accounted for majority of adverse events, followed by lymphocyte count decreased (38.1%, 16/42) and neutropenia (19%, 8/42). Hypogammaglobulinemia was observed in 30 of 42 patients. Of these, 23 were mild cases showing decreased IgG levels that did not require intervention and gradually returned to normal during follow-up. The remaining 7 patients developed more severe hypogammaglobulinemia with IgG levels below 4 g/L and required intravenous immunoglobulin replacement therapy. At the onset of neutropenia and decreased lymphocyte count, concurrent use of MMF was present in 12.5% (1/8) and 25% (4/16) episodes, respectively. No dose-dependent increase in adverse events was observed with higher rituximab doses. By the last follow-up, all adverse events were fully resolved. One patient experienced recurrent rashes during infusion and discontinued rituximab treatment. Other patients continued rituximab treatment due to these reactions by reducing rate, temporary discontinuing of infusion or adding anti-allergy medications (cetirizine hydrochloride or loratadine).

**Table 4:** Adverse events occurred in rituximab treatment in children with SD/FR nephrotic syndrome due to MCD or FSGS.

Adverse event	Total (%)
Patients with adverse events	30 (71.4%)
Fatal	0
Nonfatal	
Skin rash	1 (2.4%)
Chest tightness	1 (2.4%)
Upper respiratory tract infection	1 (2.4%)
Stomachache	1 (2.4%)
Fever	1 (2.4%)
Chill	1 (2.4%)
Flushed face	1 (2.4%)
Hypogammaglobulinemia	30 (71.4%)
Neutropenia	8 (19.0%)
Agranulocytosis	3 (7.1%)
Lymphocyte count decreased	16 (38.1%)

## 4 Discussion

In this study, we evaluated the efficacy of rituximab in patients with SDNS/ FRNS due to MCD or FSGS. Our findings revealed that rituximab proved to be an extremely effective, leading to clinical remission in all patients, significantly reducing one-year relapse rates to 35.7%, and stabilizing kidney function. During the first year of follow-up, the proportion of patients maintaining remission was more than twice that reported with corticosteroids monotherapy in historical controls (64.3% vs. 30%) (11). The adverse event profile remained acceptable, with no increase in adverse events observed after repeated courses or higher cumulative doses, supporting the repeated use of rituximab in this population.

The efficacy of rituximab in reducing relapse rates aligns with previous studies, including the NEMO study by Ruggenenti et al., which reported a significant reduction in total relapses (from 88 to 22) over one-year (12). Similarly, a retrospective study from Spain showed significantly lower annual relapse rates and higher sustained remission rates among rituximab-treated children with SDNS/FRNS (13). A meta-analysis reported a relapse rate of 35.9% in patients with MCD, and a relapse rate of 47.3% in FSGS patients treated with rituximab (14). The slightly lower relapse rate observed in our study may be attributed to the fact that our patients were steroid sensitive and in remission at the time of first received rituximab treatment. Moreover, prior exposure to other immunosuppressants may have synergistically enhanced the therapeutic response. Although the median pre-treatment relapse rate was 2 per year, treatment decisions were based not solely on relapse frequency, but also on steroid dependence, cumulative steroid toxicity, and inadequate response to second-line immunosuppressive agents.

Regarding dosing, no significant difference in relapse rates at 6 months and 1 year was observed between patients receiving 3–4 doses versus 1–2 doses during the initial course. Pharmacokinetic data on rituximab in the treatment of SDNS/FRNS remain limited. According to a single dose study (375 mg/m^2^) on 12 children with SDNS, the half-life of rituximab was 14.5 days (15). Another study involving 14 children receiving one or two rituximab doses showed a half-life of 11.7 days (16). A further study of 48 Japanese children receiving four weekly 375 mg/m^2^ doses, reported a half-life of 23 days (17). Proteinuria has been proposed as a key factor contributing to shortened rituximab half-life, rather than the number of infusions administered in the first course (18, 19). In addition, one study reported the patients with a remarkably short half-life exhibited persistent negative proteinuria (16). Given that response improvement does not appear to be dose-dependent, and that lower cumulative doses are associated with reduced economic burden and fewer side effects, we suggest that 1–2 doses of rituximab may be sufficient in the initial course (20).

Histological subtype has not been identified as a predictive factor of relapse (21). In our cohort, the relapse times did not differ significantly between MCD and FSGS, consistent with findings in adults by Hansrivijit et al. and in children by Hansrivijit et al. (14) and Sinha et al. (22). Notably, FSGS patients exhibited superior 96-month relapse-free survival compared to MCD patients. This indicates that steroid sensitive FSGS patients respond well to rituximab therapy. However, given the limited number of FSGS patients (*n* = 6) in our cohort, this observation should be interpreted with caution and requires confirmation in larger, prospective multicenter studies. These results highlight the potential of rituximab as a valuable treatment option for patients with SDNS/FRNS due to FSGS, especially those who have failed other immunosuppressive therapies.

Although FRNS is often considered clinically milder than SDNS, treatment decisions in our study were not based solely on disease classification. Many FRNS patients still experienced a high relapse burden, requiring repeated courses of corticosteroids and prolonged exposure to steroid therapy. In addition, several patients had previously received steroid-sparing immunosuppressive agents, including calcineurin inhibitors or mycophenolate mofetil. Under these circumstances, rituximab was used as a steroid-sparing strategy to reduce relapse frequency and cumulative treatment toxicity. According to the KDIGO 2025 clinical practice guideline, rituximab can be considered in children with SDNS or FRNS who have frequent relapses or who experience adverse effects from conventional immunosuppressive therapies.

Though rituximab is safe in children, it is important to note that serious complications, including fatal hepatitis reactions and fulminant myocarditis, can indeed occur (6, 23, 24). Previous studies did not indicate that a higher total dosage or increasing treatment courses resulted in more side effects (20, 23, 25). Our data support these findings, indicating that hypogammaglobulinemia, neutropenia, and a reduction in lymphocyte count were the three most frequently observed adverse events. Worth to be mentioned, the number of patients with infection complications in our study was lower than previously reported (26). This difference may be attributed to the lower dose of rituximab used (375 mg/m^2^ every time). Moreover, infusion of gamma globulin as appropriate to patients with hypogammaglobulinemia may be another reason for reducing infection.

This study has several limitations, including its retrospective, single-center design and the small sample size, particularly in the FSGS subgroup. The limited number of FSGS patients resulted in an imbalanced group size, which may have compromised the stability and generalizability of our statistical estimates. Consequently, the findings may not be fully applicable to broader patient populations. To validate our results and to optimize rituximab treatment protocols for SDNS/FRNS, future prospective, multicenter randomized controlled trials with larger cohorts and longer follow-up are warranted.

In conclusion, rituximab represents an effective and well-tolerated treatment option for children with SDNS/ FRNS caused by MCD or FSGS. Rituximab therapy achieved high rates of clinical remission, significantly reduced relapse frequency, and enabled a substantial reduction in dependence on corticosteroids and other immunosuppressive agents, thereby improving long-term outcomes. Its safety profile remained acceptable even after repeated courses. Side effect profiles remain acceptable even after multiple courses of rituximab. The encouraging results clearly support repeated rituximab use in children with SDNS/FRNS. However, due to the small number of FSGS patients included, the observed difference in relapse-free survival between FSGS and MCD should be interpreted with caution and warrants further investigation in larger studies. There is a need for prospective trials to investigate the immunological alterations and determine the best treatment strategy for this group of patients.

## Data Availability

The original contributions presented in the study are included in the article/Supplementary material, further inquiries can be directed to the corresponding authors.
