# Supplementary material for: The efficacy of rituximab in pediatric patients with steroid-dependent or frequent relapsing nephrotic syndrome due to MCD or FSGS
Source: Front Med (Lausanne). 2026 Apr 23;13:1763615. doi: 10.3389/fmed.2026.1763615 (PMC13149059; doi:10.3389/fmed.2026.1763615)
Supplement: Supplementary file 1 [file Supplementary_file_1.docx]

Supplementary Figure S1

Relapse times comparisons in children with SD/FR nephrotic syndrome with different doses of rituximab in the first course. (A) children received 4 doses of rituximab and those received 1-3 doses, 6 months after the first rituximab treatment; (B) children received 4 doses of rituximab and those received 1-3 doses, one year after the first rituximab treatment; (C) children received 3-4 doses of rituximab and those received 1-2 doses, 6 months after the first rituximab treatment; (D) children received 3-4 doses of rituximab and those received 1-2 doses, one year after the first rituximab treatment.

Supplementary Figure S2

Rituximab infusion status comparisons between MCD patients and FSGS patients. (A) total times of Rituximab infusion during all the courses; (B) total courses of Rituximab infusion during overall follow-up time.

Supplementary Figure S3

Relapse times comparisons between MCD patients and FSGS patients after the first course of rituximab treatment. (A) 6 months after the first course of rituximab treatment; (B) one year after the first course of rituximab treatment.
